# Sirolimus in Refractory Cronkhite-Canada Syndrome and Focus on Standard Treatment

**DOI:** 10.1177/2324709618765893

**Published:** 2018-03-22

**Authors:** Catherine Langevin, Hugo Chapdelaine, Jean-Maxime Picard, Pierre Poitras, Raymond Leduc

**Affiliations:** 1 Centre Hospitalier de l’Université de Montréal, Montreal, Quebec, Canada; 2 Montreal Clinical Research Institute, Montreal, Quebec, Canada; 3 Chicoutimi Hospital, Chicoutimi, Quebec, Canada

**Keywords:** Cronkhite-Canada syndrome, sirolimus, treatment

## Abstract

Cronkhite-Canada syndrome is a rare syndrome consisting of extensive gastrointestinal polyposis and ectodermal changes including cutaneous hyperpigmentation, alopecia, and onychodystrophy. We report the case of a 45-year-old Caucasian male patient who failed multiple treatments over 2 years including steroids, azathioprine, adalimumab, and cyclosporine. He had recurrent and prolonged hospitalizations because of diarrhea, abdominal pain, weight loss, and malnutrition. Sirolimus was initiated with a significant clinical and endoscopic benefit apparent within, respectively, 2 and 8 weeks. An ongoing remission was achieved and maintained for over 6 months after prednisone tapering. We review the current evidence on treatment of Cronkhite-Canada syndrome and suggest the incorporation of sirolimus in that algorithm.

Cronkhite-Canada syndrome (CCS) is a rare noninherited condition characterized by diffuse gastrointestinal hamartomatous polyposis and ectodermal changes including cutaneous hyperpigmentation, alopecia, and onychodystrophy. Main complaints are diarrhea, abdominal pain, and weight loss. Mean age at onset is 59 years. Microscopic examination regularly shows inflammatory cell infiltration with mononuclear cells and eosinophils. Etiology remains uncertain, but immune dysregulation plays a large role. Prognosis in CCS remains poor with a 5-year mortality of 55%.^1^ Colorectal and gastric cancers have been reported in, respectively, 8% (31/374) and 5% of CCS patients,^2^ but patients in remission for >3 years had a lower risk. We report the case of a 45-year-old man with diarrhea, abdominal pain, weight loss, and malnutrition who was diagnosed with CCS and resisted to a panoply of treatments before finally responding to sirolimus.

A 45-year-old Caucasian man presented in September 2015 with abdominal pain, diarrhea, weight loss, dysgeusia, alopecia, and brownish skin changes. He was treated for cellulitis a few weeks ago and was otherwise healthy. He underwent upper and lower endoscopies, which revealed nodular antral infiltration, villous atrophy, and numerous polyps in the colon. He was diagnosed with CCS and put on prednisone 50 mg/day, which induced an initial clinical response. Azathioprine 75 mg/day was added but had to be discontinued due to hepatic cytolysis. The disease remained steroid-dependent despite adalimumab 40 mg every 2 weeks and then every week. This treatment was interrupted after 9 months because of disease progression with worsening gastric infiltration and colonic polyps up to 3 cm. Cyclosporine (aiming for ≥200 ng/mL levels) was introduced without response after 2 months. In January 2017, the patient was hospitalized for severe malnutrition, weight loss, and incapacity to eat due to gastric outlet obstruction by a polypoid antral mass. When transferred at our center in February 2017, his treatment included parenteral nutrition, cyclosporine, prednisone, cotrimoxazole 3×/week, iron, calcium, and vitamins K and D 1×/wk. On admission, he had microcytic anemia (93 g/L), hypoalbuminemia (17 g/L), vitamin D deficiency (22 nmol/L), low ferritin (9.4 µg/L), low immunoglobulin G (IgG), low selenium, and elevated fecal calprotectin (>2100 µg/g, normal <50). Creatinine, liver function tests, vitamins B_12_/A/E, folic acid, copper, manganese, IgA, IgM, C-reactive protein, *Helicobacter pylori* serum antibody, procalcitonin, and IgG4 were within normal limits. Autoimmune workup was negative. Endoscopy (Figure 1) showed numerous polyps covering the gastric mucosa, some large ones forming a pseudomass almost completely obstructing the antral lumen. The duodenum was carpeted with multiple flat polyps less than 1 cm in diameter. The colon was diffusely covered by multiple small hamartomous-like polyps. Biopsies for *H pylori* were negative. IgG4-positive-cell count/high-power field was modestly increased (19 cells per field). Methylprednisolone 20 mg intravenous 3×/d was started on arrival and cyclosporine was continued. Facing poor clinical response, cyclosporine was switched to sirolimus (4-6 mg 1×/day aiming for blood levels 8-10 ng/mL). Prednisone 50 mg/day initially (following intravenous methylprednisolone) was progressively decreased. Frank improvement was noted at week 2 on sirolimus with resolution of abdominal pain and progressive restoration of the capacity to eat although diarrhea (400-800 g per day) reappeared with oral feeding. At week 4, prednisone was weaned off completely without any flare-up. At week 8, the patient had gained 5 kg, early upper endoscopy assessed partial healing, and the patient was finally discharged from the hospital with home parenteral nutrition (decreased from 7 to 5 nights per week), sirolimus 5 mg 1×/day, and free diet. Parenteral nutrition was stopped at week 12 as the patient could eat a normal oral diet. Dysgeusia and asthenia disappeared, and he had only 3 to 4 stools per day. Hemoglobin and serum albumin were normalized. Six months later, he had regained a normal life. He was back to work and returned to playing hockey. After 8 months of sirolimus, upper and lower endoscopies showed a decrease in the number and size of the polyps (Figure 2).

**Figure 1.**
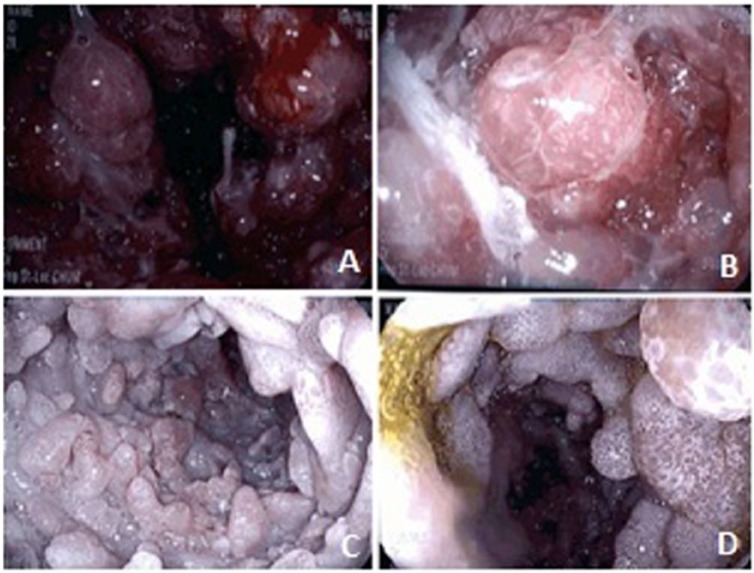
Endoscopic appearance of stomach (A, B), duodenum (C), and colon (D) at arrival at our center.

**Figure 2.**
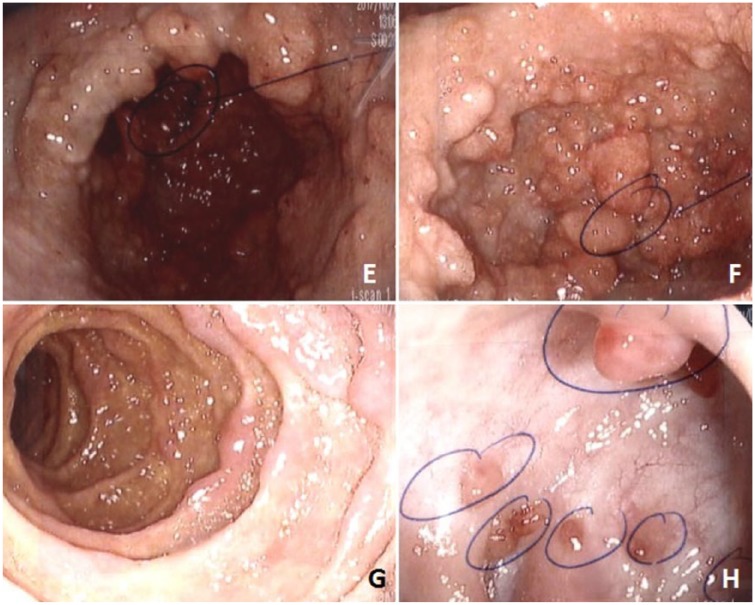
Endoscopic appearance of stomach (E, F), duodenum (G), and colon (H) after 8 months of sirolimus.

We report the case of a patient with CCS refractory to all suggested treatments who achieved impressive clinicoendoscopic remission and maintenance when treated with sirolimus. Current standard treatment of CCS is based on the 500+ cases reported in the literature. All patients should receive aggressive nutritional support and parenteral nutrition as needed. Corticosteroids induced and maintained remission in an important proportion of the reported cases with prednisone 40 mg/day being the recommended dose.^3^ They should be slowly tapered and discontinued when a sustained response is obtained. Azathioprine maintained remission without relapse in 5 patients of the Mayo Clinic for a median of 4.5 years.^4^ It is the most recognized steroid-sparing agent in CCS. As for anti–tumor necrosis factor drugs, high tissue tumor necrosis factor-α level was reported. Infliximab induced complete remission and maintenance in one case.^5^ Infiltration by IgG4-positive plasma cells was also present in half of examined CCS polyps^6^ to a lesser extent than in IgG4-related disease, but to our knowledge, no experience was reported with a B-cell depletion therapy such as rituximab. Other treatments reported in the literature include various combinations of histamine-receptor inhibitor, proton pump inhibitor, mesalamine, sulfasalazine, antibiotics, antiplasmin agent, and cromolyn sodium.^1,3^ Treatment of *H pylori* was favored when present as remissions were reported after eradication.^7^ Surgery (subtotal gastrectomy, large bowel resection) has been used to improve hypoproteinemia in some cases. Calcineurin inhibitors repress T-cell activation by blocking interleukin-2 production. Success was reported in 2 patients on cyclosporine and 1 patient on tacrolimus.^8-10^ mTOR inhibitors block interleukin-2 response, but to our knowledge, experience in CCS is lacking. Arguments in favor of using sirolimus in our patient with refractory CCS were the following: (1) sirolimus (rapamycin) 5 ng/mL^11^ and everolimus^12^ have been successfully used in adult refractory inflammatory bowel disease; (2) it also led to positive outcomes in 10/14 pediatric patients with refractory inflammatory bowel disease^13^; (3) and mostly, sirolimus reduced the quantity and size of polyps in Peutz-Jeghers syndrome mouse model, possibly via an antiangiogenic effect.^14^ In the present case report, sirolimus provided significant clinical and endoscopic benefit apparent within, respectively, 2 and 8 weeks, with an ongoing remission of 6+ months in a patient who failed multiple treatments over 2 years including steroids, azathioprine, adalimumab, and cyclosporine. We suggest that sirolimus could be a valuable therapeutic option for CCS patients.
